# Air–Liquid–Solid Triphase Interfacial Microenvironment Regulation for Efficient Visible-Light-Driven Photooxidation Based on Ordered TiO_2_ Porous Films

**DOI:** 10.3390/biomimetics11040261

**Published:** 2026-04-10

**Authors:** Lijun Zhou, Zhaoyue Tan, Xia Sheng, Xinjian Feng

**Affiliations:** 1 State Key Laboratory of Bioinspired Interfacial Materials Science, College of Chemistry, Chemical Engineering and Materials Science, Soochow University, Suzhou 215123, China; 2 Suzhou Institute for Advanced Research, University of Science and Technology of China, Suzhou 215123, China

**Keywords:** interfacial microenvironment, air–liquid–solid triphase system, photooxidation, hydrophobic molecules

## Abstract

The rational design and regulation of interfacial microenvironments represents an effective strategy for enhancing reaction performance. Previous studies have demonstrated that constructing air–liquid–solid triphase interfaces can substantially enhance catalytic reactions involving gaseous reactants. However, research on regulating the triphasic interfacial microenvironment remains limited and challenging. Herein, we fabricated a triphase photocatalytic system by depositing hydrophobic materials onto ordered TiO_2_ porous (OTP), achieving significantly enhanced performance in visible-light-driven dye-sensitized photooxidation. Further, we regulated the triphasic microenvironment by systematically adjusting the chain length of hydrophobic molecules. It was found that the chain length greatly affects the interfacial properties, including O_2_ concentration, the organic molecule adsorption and the interfacial electron transfer efficiency, thereby influencing photocatalytic reaction kinetics and pathways. We demonstrated a high-performance triphase photocatalytic system using 1H,1H,2H,2H-perfluorooctyl triethoxysilane as the hydrophobic material, which optimized multiple interfacial properties through synergistic effects, leading to optimal photocatalytic performance.

## 1. Introduction

Nature is a rich source of inspiration for scientific and technological innovation. In recent decades, by mimicking natural non-wetting surfaces such as lotus leaves and water strider legs, artificial materials with various special wetting properties have been developed [1,2,3,4] for numerous applications, including antifouling techniques [5], lubrication [6], microfluidic operation [7], water–oil separation [8], and fog collection [9]. In addition to biomimetic morphology, bioinspired non-wetting techniques have also been used to improve chemical reaction performance [10,11,12]. When a solid with special wettability comes into contact with a liquid, unique multiple-phase coexisting interfaces are created, such as gas–liquid–solid interfaces and oil–water–liquid interfaces [12,13,14,15,16]. This offers an opportunity to engineer the interfacial microenvironment where chemical reactions take place.

The reaction interface microenvironment in a heterogeneous catalysis significantly influences the catalytic process and kinetics and is now recognized as being equally important to catalytic material optimization [17,18,19,20]. The interfacial microenvironment around the catalyst often affects the diffusion of reactants and products, the local pH value, and the stability of active sites [21,22,23,24]. Changing a liquid–solid diphasic interface to a gas–liquid–solid triphasic interface can increase the concentration of gaseous reactants at the interface, thereby boosting the kinetics and/or selectivity of photocatalytic, enzymatic and electrocatalytic reactions [24,25,26,27,28,29,30]. Enhancing the hydrophobicity of catalysts by mimicking natural non-wetting surfaces is an effective strategy to construct triphasic interfaces [26,31], as the hydrophobic layer can trap gas on the catalyst surface. However, current studies mainly focus on the construction of triphase systems, while research on regulating the triphasic microenvironment and elucidating how the hydrophobic layer governs interfacial properties remains limited and challenging.

Dye-sensitized semiconductor-mediated photooxidation is a green approach that uses visible light to decompose toxic dyes [32,33,34,35,36,37]. Dye molecules absorbed on the semiconductor are excited by visible light and inject electrons into the conduction band (CB) of the semiconductor; O_2_ then reacts with these CB electrons and generates reactive oxygen species (ROS) for dye oxidation [33]. In a conventional photocatalytic system, the photooxidations occur at a liquid (water)–solid (semiconductor) (L–S) diphase interface, which is not conducive to the enrichment of O_2_ and adsorption of organic dye molecules at the interface. It often suffers from poor performance both in photocatalytic kinetics and mineralization [19,21,26].

Very recently, we constructed an efficient triphase interface photocatalytic system based on a three-dimensional ordered TiO_2_ porous (OTP) film via depositing a thin layer of polydimethylsiloxane (PDMS), which is a commonly used hydrophobic material with excellent hydrophobicity and thermal stability, onto the OTP surface. This photocatalytic system creates an air–liquid–solid triphasic interface microenvironment with high concentrations of both O_2_ and organic dye molecules, leading to a much-enhanced photocatalytic performance [38]. However, the PDMS layer is a hydrophobic material with relatively long molecular chains. This hinders the electron transfer between the dye and TiO_2_, thereby affecting the photocatalytic performance.

Herein, we constructed triphase systems based on ordered TiO_2_ porous (OTP) films and tuned the interfacial microenvironment by varying the chain length of hydrophobic molecules. The correlation between the chain length of the molecular and interfacial properties, including the interfacial O_2_ concentration, the organic molecule adsorption, and the electron transfer, was systematically investigated. It was found that the triphase system with deposited 1H,1H,2H,2H-perfluorooctyl triethoxysilane (Octyl-TES) thin film exhibits the synergic advantages of high interfacial O_2_ concentration, high organic adsorption capacity, and efficient electron transfer, which collectively enhance the performance of dye-sensitized photooxidation.

## 2. Materials and Methods

### 2.1. Chemicals and Reagents

Ethanol (G.R.), hydrochloric acid (G.R.), and cyclohexane (A.R.) were purchased from Sinopharm Chemical Reagent Co., Ltd., (Shanghai, China). 1H,1H,2H,2H-perfluorohexyl triethoxysilane (Hexyl-TES, G.C.), 1H,1H,2H,2H-perfluorooctyl triethoxysilane (Octyl-TES, G.C.), 1H,1H,2H,2H-perfluorodecyl triethoxysilane (Decyl-TES, G.C.), 1H,1H,2H,2H-perfluorododecyl triethoxysilane (Dodecyl-TES, G.C.), and Rhodamine B (≥99%) were purchased from Macklin. 5,5-dimethyl-1-pyrroline N-oxide (DMPO, ≥97%) and tetraethoxysilane (TEOS, ≥97%) were purchased from Aladdin. Titanium dihydroxybis (ammonium lactate) (IV) (TiBALDH, 50% *w*/*w* aqueous solution) was purchased from Alfa Aesar. Polydimethylsiloxane (PDMS, vinyl end seal, molecular weight 6000 Da) was purchased from Gelest. All solutions were prepared using ultrapure distilled water (18.2 MΩ cm, Hitech laboratory water purification system).

### 2.2. Preparation of Three-Dimensional Ordered TiO_2_ Porous (OTP) Film

Preparation of Ti and Si precursors was carried out. Si precursor: The solution consisted of TEOS, with a volume ratio of 1:1:2, 0.1 M hydrochloric acid, and ethanol, and was magnetically stirred at room temperature for 1 h. Ti precursor: The solution consisted of dihydroxybis (ammonium lactate) TiBALDH in a volume ratio of 1:5 with 0.1 M hydrochloric acid, and it was magnetically stirred at room temperature for 1 h. Preparation of OTP film: The OTP film was synthesized using the template method with PS spheres with a diameter of 290 nm as the template. The prepared PS spheres were diluted to 60 mg/L in an aqueous solution, and the diluted PS spheres were thoroughly mixed with the precursor sol of 150 μL with a Si-Ti molar ratio of 3:7. The dosage of the Si precursor and Ti precursor in each mL of Si-Ti precursor was calculated using the formula 7.5 × (x) mL (Si precursor) and 12 × (1 − x) mL (Ti precursor), where x is between 0 and 1. Place the mixture on the magnetic stirrer and mix it for 12 h. Apply different amounts of evenly mixed droplets to the clean, ordinary glass base (fixed area: 0.7 × 2 cm^2^). Then, place the sample in a chamber with a constant temperature of 45 °C and 90% humidity for 3 h to evaporate the solvent. The resulting sample is calcined in a tube furnace in an oxygen atmosphere to remove the PS ball template. The calcination process is as follows: first increase the temperature to 300 °C at a heating rate of 2 °C/min and maintain for 2 h; then increase from 300 °C to 500 °C at 1 °C/min and maintain for another 2 h; and finally allow it to cool naturally to room temperature.

### 2.3. Deposition of Hydrophobic Layers

The prepared OTP film was immersed in a cyclohexane solution containing an appropriate amount of hydrophobic material (Hexyl-TES, Octyl-TES, Decyl-TES and Dodecyl-TES) at room temperature for 2 h. Typically, the concentration of hydrophobic material/cyclohexane solution is 10% by volume. Subsequently, the film was washed with cyclohexane 5 times, and then dried in air until the cyclohexane was completely evaporated. Finally, heat the film in an oven at 120 °C for 1 h to solidify the hydrophobic layer. The deposition of the PDMS layer was achieved by soaking the as-prepared OTP film in PDMS for 10 min. The film was then washed with cyclohexane 5 times and allowed to dry in the air. Finally, the film was irradiated with 20 mW/cm^2^ ultraviolet light with a wavelength of 367 ± 5 nm for 5 min.

### 2.4. Characterization

The morphologies of the OTP and hydrophobic material-deposited OTP (H-OTP) were characterized using scanning electron microscopy (SEM, S4700, Hitachi, Tokyo, Japan) with an energy-dispersive spectrometer (Oxford EDS, Regulus 8230, Hitachi, Tokyo, Japan). And a transmission electron microscope (TEM, Tecnai G20 F20 S-Twin, USA) was used. For ROS radical analysis, an electron spin resonance spectrometer (ESR, JES-X320, Japan) was used. The experiments were performed under a solar simulator (light intensity: 100 mW cm^−2^) at room temperature. The Mod Width value was set to 0.1 mT, and the amplitude was 600. DMPO was used for hydroxyl radical trapping. The surface hydrophobicity was characterized using water contact angle measurements (JC2000D6, Powereach, China). The contacting behavior between H-OTP and dye solution was examined by using a three–dimensional laser scanning confocal microscope (LSCM, LSM 800 with airyscan, Germany). OTP and H-OTP were immersed in an RhB solution (10 mg L^−1^) for 15 min, taken out, and dried naturally. The fluorescent signal of RhB molecules adsorbed on the surface was recorded using LSCM with a 63-fold oil lens. The three-dimension cross-sectional fluorescence reconstruction was obtained by serial layer scanning of the porous structure along the Z-axis. The oxygen concentrations in aqueous solutions were measured with a portable dissolved oxygen meter (Sanxin MP516, China). The dissolved oxygen meter was placed in the water after adding 6 mL of the solution to the beaker. After the meter had been stabilized for 2 min, the sample was added to the aqueous solution, while the meter continuously monitored the dissolved oxygen level.

### 2.5. Visible-Light-Driven Photocatalytic Oxidation Experiments

The visible light catalytic reaction was carried out in a 4.5 mL quartz cell, degrading 2 mL Rhodamine B (RhB). A Microsolar 300 Xe lamp (100 mW cm^−2^) was used as a solar simulator, with an ultraviolet filter (λ > 420 nm) being used as the light source. A stirring rate of 500 rpm was employed during the measurements. A UV-Vis spectrophotometer (EVOLUTION 220, Thermo, USA) was used to monitor the change in the absorption spectrum of each dye solution at 15 min intervals to calculate the residual RhB concentration. These experiments were carried out under ambient temperature.

## 3. Results and Discussion

The three-dimensional ordered TiO_2_ porous (OTP) film was synthesized by a template method as reported in our previous study [38]. Fabrication of the triphase photocatalytic system based on the OTP film involves depositing a layer of hydrophobic molecules (as detailed in the experiment) to form a hydrophobic OTP film (H-OTP). The hydrophobic materials used in this study include 1H,1H,2H,2H-perfluorohexyl triethoxysilane (Hexyl-TES), 1H,1H,2H,2H-perfluorooctyl triethoxysilane (Octyl-TES), 1H,1H,2H,2H-perfluorodecyl triethoxysilane (Decyl-TES), 1H,1H,2H,2H-perfluorododecyl triethoxysilane (Dodecyl-TES) and polydimethylsiloxane (PDMS).

**Figure 1:**
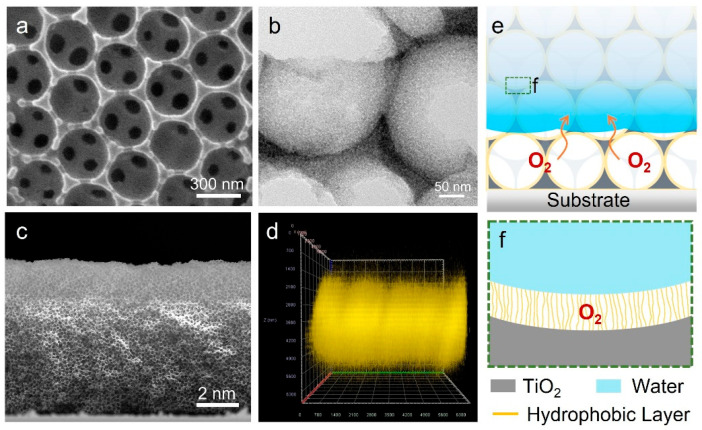
(**a**) Scanning electron microscope (SEM) image of the OTP@Octyl-TES film. (**b**) Transmission electron microscopy (TEM) image of the OTP@Octyl-TES film. (**c**) Cross-sectional SEM image of the OTP@Octyl-TES film. (**d**) Three-dimensional laser scanning confocal microscopy fluorescence image of RhB molecules adsorbed on OTP@Octyl-TES film. (**e**) Schematic of the OTP@Octyl-TES film in contact with RhB solution. (**f**) Enlarged view of the interface microenvironment.

Figure 1a,b show the typical scanning electron microscopy (SEM) and the transmission electron microscope (TEM) image of the H-OTP film with an Octyl-TES layer (OTP@Octyl-TES). It consists of multilayered microporous frames with regularly arranged macropores that have a diameter of about 300 nm. The high-resolution TEM image (Figure S1a) shows a clear fringe with fringe spacing of 0.352 nm, corresponding to anatase TiO_2_ (101). As shown in Figure S1b, an amorphous layer with a thickness of about 1 nm can be observed on the TiO_2_ surface, representing the presence of a hydrophobic molecule layer. The uniform distribution of Ti and F elements in the energy-dispersive X-ray spectroscopy (EDS) (Figure S2) also revealed the presence of an Octyl-TES layer. After hydrophobic molecule modification, the contact angle (CA) changed from 10° to 137° (Figure S3), indicating that the surface wettability changed from hydrophilic to hydrophobic.

The as-synthesized OTP@Octyl-TES film was then characterized using 3D laser scanning confocal microscopy (LSCM) to investigate the contact behavior between the OTP@Octyl-TES film and the dye solution. Before the measurement, the OTP@Octyl-TES film was immersed in a Rhodamine B (RhB) solution for 15 min, and then removed and dried naturally. In this case, RhB molecules will adsorb on the area where the film is in contact with the solution. Figure 1e shows the cross-sectional view of a 3D LSCM fluorescence image. The bright region, which represents the presence of dye molecules, is about 4 μm. This indicates that the dye solution can wet the top 4 μm of the film. As the total thickness of the film is about 7 μm (Figure 1c), the solution cannot wet the bottom 3 μm of the film. In this case, as shown in Figure 1e, air can be transported through the unwetted pores to the interface, forming an air–liquid–solid triphase interface. The schematic diagram of an enlarged view of the interface microenvironment is shown in Figure 1f. Hydrophobic molecules enable a layer of air to be trapped on the surface of TiO_2_, forming an O_2_-rich microenvironment at the interface.

**Figure 2:**
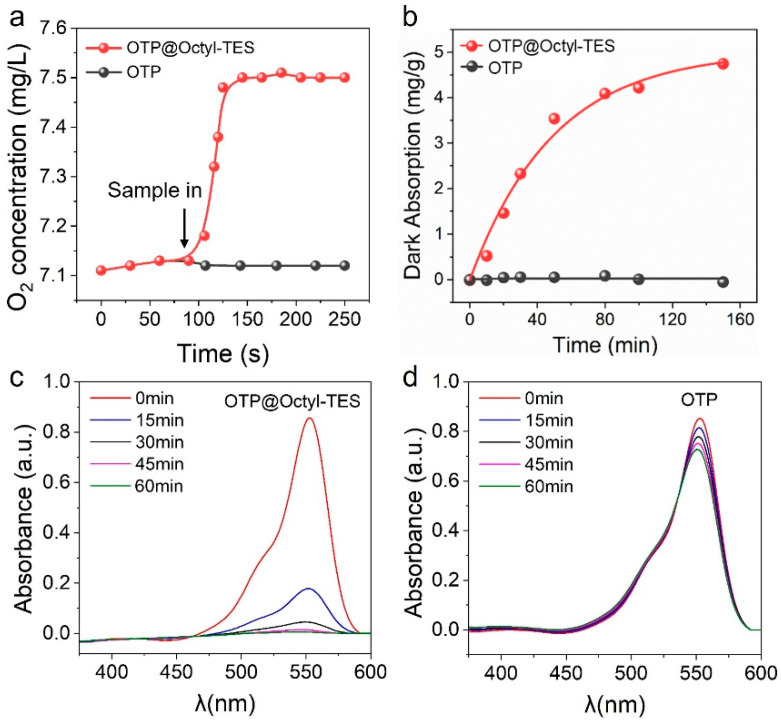
(**a**) Dynamic O_2_ concentration in water after immersion of OTP@Octyl-TES and OTP films. (**b**) Adsorption kinetics of RhB molecules based on OTP@Octyl-TES and OTP films. Absorption spectra of the RhB solutions as a function of illumination time based on OTP@Octyl-TES film (**c**) and OTP film (**d**) in visible light-driven dye-sensitized photooxidation.

The triphase photocatalytic system based on OTP@Octyl-TES film was then applied in a dye-sensitized self-oxidation under visible light (λ > 420 nm). Rhodamine B (RhB) was chosen as a model molecular in this work. First, the interfacial properties were compared between the triphase and conventional diphase system. As seen in Figure 2a, when the OTP@ Octyl-TES film is immersed in water, a significant increase in O_2_ concentration in the water can be observed. This is due to O_2_ diffusing from the air–liquid–solid triphase interface into the bulk water, which indicates that the triphase system has a high O_2_ concentration at the interface. Figure 2b compares the dye absorption properties of untreated OTP and OTP@Octyl-TES film. Previous studies have reported that the presence of a hydrophobic layer on the TiO_2_ surface benefits the organic adsorption at the interface [16,39]. On hydrophilic TiO_2_ surfaces (conventional diphase interface), RhB adsorption proceeds via esterification between the dye’s carboxylic groups and surface hydroxyl groups. However, this process must overcome the hydration layer barrier, with RhB competing against water for hydrophilic binding sites. In sharp contrast, hydrophobic TiO_2_ surfaces (triphase interface) enable direct interaction between the hydrophobic layer and the hydrophobic ethyl groups of RhB, facilitating disruption of hydration films and markedly enhancing the adsorption capacity. Therefore, the OTP@Octyl-TES film exhibits significantly higher adsorption capacity of RhB than OTP film.

In a dye-sensitized self-oxidation process, the kinetics commonly follows a pseudo-first-order kinetic with the apparent first-order reaction rate being constant $K_{app}=kC_{O2}^{\mathrm{int}}K_{RhB}$, where $C_{O2}^{\mathrm{int}}$ is the concentration of O_2_ in the reaction interface microenvironment, *K*_RhB_ is the Langmuir adsorption constant of RhB, and *k* is the reaction rate constant [26]. Based on the triphase system, both the interfacial O_2_ concentration and dye adsorption capacity is improved, and thus the photocatalytic performance is greatly enhanced. As seen in Figure 2c,d and Figure S4a, the degradation rate of RhB in the OTP@Octyl-TES system reaches 98%, whereas it is only 11% in the conventional diphase system. The kinetics plot is seen in Figure S4b. Both OTP and OTP@Octyl-TES systems follow a pseudo-first-order kinetic. The $K_{app}$ of OTP@Octyl-TES system is calculated as 0.218 min^−1^, which is over 50-fold higher than that of the OTP system.

In our previous study, we used PDMS, which is a commonly used hydrophobic material with excellent hydrophobicity and thermal stability, to construct a triphase system, and also obtained significantly enhanced performance in comparison to diphase systems [38]. Figure S5 compares photocatalytic performances of the OTP@Octyl-TES and OTP@PDMS systems. After a 1 h reaction, the RhB absorption peak disappeared almost completely in the OTP@Octyl-TES system without any significant blue shift. In contrast, in the OTP@PDMS system, the RhB absorption peak undergoes a significant blue shift, resulting in a slower reaction rate of the system after the blue shift. This demonstrates that the photooxidation undergoes different reaction pathway in these two systems.

In a typical dye-sensitized self-oxidation process, after being excited, the dye injects electrons into CB of TiO_2_. The CB electrons react with O_2_ and will generate •O_2_^−^ first. •O_2_^−^ can be captured by RhB molecules, and can also convert to hydroxyl radicals (•OH) through chain reactions as follows [21,32]:

(1) •O_2_^−^ + RhB → RhB*

(2) $$•{O_{2}}^{-}+2H^{+}+e_{CB}^{-}\to H_{2}O_{2}$$

(3) $$H_{2}O_{2}+e_{CB}^{-}\to •OH+OH^{-}$$

**Figure 3:**
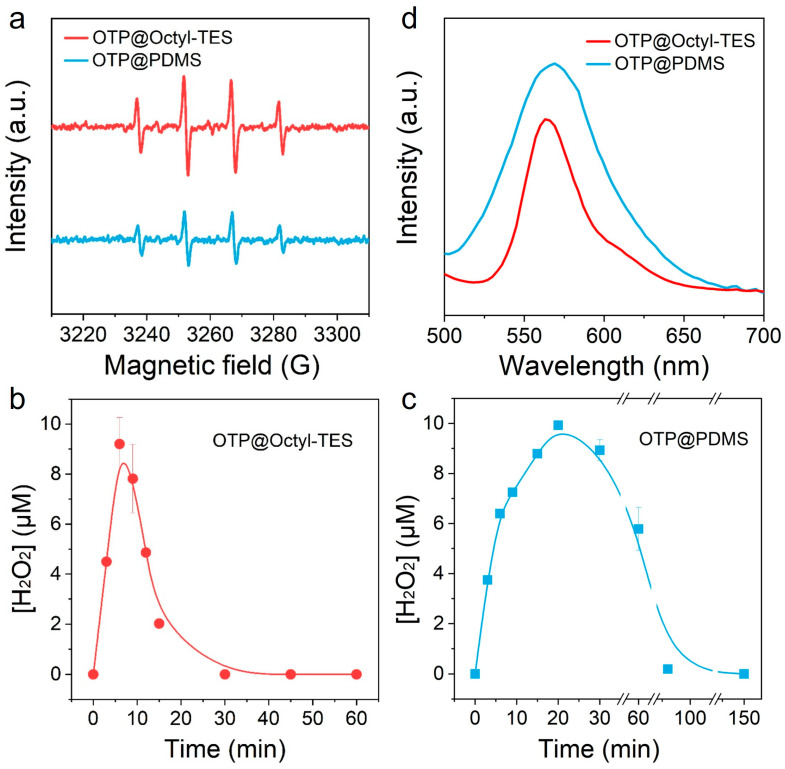
(**a**) Electron spin resonance spectrometer (ESR) of DMPO-•OH of OTP@Octyl-TES and OTP@PDMS system. H_2_O_2_ concentration during the reaction of OTP@Octyl-TES (**b**) and OTP@PDMS (**c**) system. (**d**) Photoluminescence (PL) spectra of OTP@Octyl-TES and OTP@PDMS system.

The blue shift in the RhB absorption peak is attributed to the oxidation of RhB by •O_2_^−^ (reaction 1) [40]. When the absorption peak shifts to approximately 498 nm, •O_2_^−^ with low oxidation capacity cannot continue to oxidize and requires •OH, which has a higher oxidation capacity. We investigated the •OH through electron spin resonance spectrometer (ESR) measurements. 5, 5-dimethyl-1-pyrroline N-oxide (DMPO) in deionized water was used for •OH trapping [41]. As seen in Figure 3a, compared to the OTP@PDMS system, the OTP@Octyl-TES system generates a higher concentration of •OH. Further experiments investigated the H_2_O_2_ concentration during the process in these two systems. According to reactions 2 and 3, •O_2_^−^ reacts with electrons to generate H_2_O_2_, which subsequently decomposes to generate •OH. As shown in Figure 3b,c, in both systems, rapid generation and accumulation of H_2_O_2_, followed by its decomposition, was observed. In the OTP@Octyl-TES system, the accumulation of H_2_O_2_ reaches its maximum value after 6 min of reaction, and then rapidly decomposes and reaches an equilibrium state (dynamic equilibrium between generation and decomposition) after about 30 min. In contrast, in the OTP@PDMS system, the H_2_O_2_ concentration reaches its maximum after 20 min of reaction, and reaches an equilibrium state after 90 min. This indicates that the OTP@Octyl-TES system has fast generation and decomposition rates of H_2_O_2_, enabling it to efficiently generate •OH in a relatively short time. A high concentration of •OH at the start of the reaction allows it to oxidize RhB directly, thus avoiding the obvious blue shift in the RhB absorption peak.

The interfacial properties of the OTP@Octyl-TES and OTP@PDMS systems were characterized. As shown in Figure S6, the interfacial O_2_ level and dye adsorption capacity are comparable. Compared to Octyl-TES, PDMS is a long-chain polymer with the molecular formula C_2_H_3_(CH_3_)_2_SiO(Si(CH_3_)_2_O)_n_Si(CH_3_)_2_C_2_H_3_. It may cause steric hindrance, thereby inhibiting the injection efficiency of photogenerated electrons from dyes into the CB of TiO_2_ [42]. We then conducted photoluminescence (PL) experiments to investigate the electron transfer property. Upon light illumination, RhB will be excited and generate photoelectrons. If electron transfer takes place, the PL intensity will decrease [43]. As shown in Figure 3d, under the condition of comparable dye adsorption, the PL intensity of OTP@PDMS is obviously higher than that of OTP@Octyl-TES. This indicates that the OTP@Octyl-TES system is more conducive to the electron transfer, which suppresses the recombination of electrons with dye cations, forming a higher interface electron density. This further promotes the generation of •O_2_^−^ and its reaction with electrons, increasing the generation efficiency of •OH.

**Figure 4:**
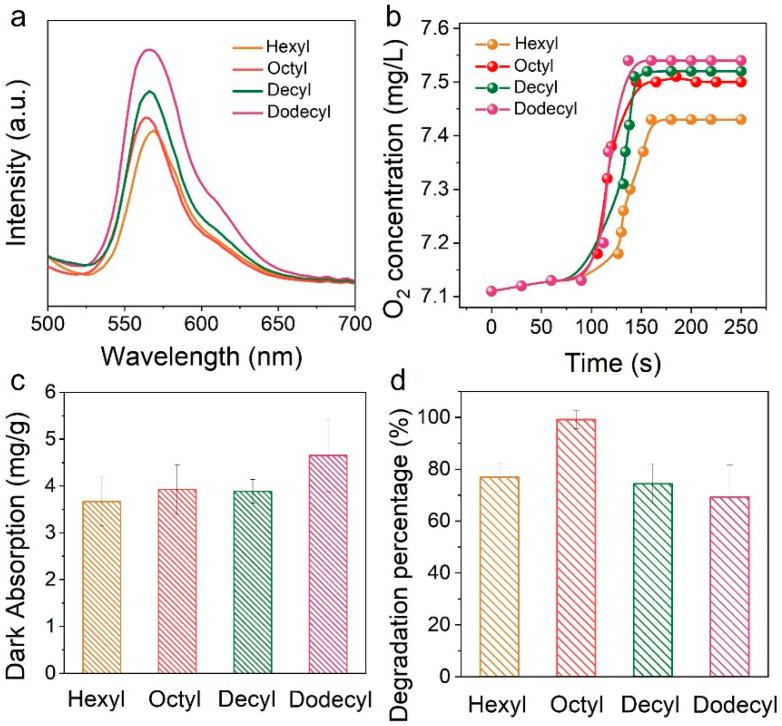
(**a**) PL spectra of OTP@Hexyl-TES, OTP@Octyl-TES, OTP@Decyl-TES and OTP@Dodecyl-TES systems (**b**) Dynamic O_2_ concentration in water after immersion of OTP@Hexyl-TES, OTP@Octyl-TES, OTP@Decyl-TES and OTP@Dodecyl-TES films. (**c**) Adsorption quantity after 1 h of dark adsorption reaction based on the four films. (**d**) Photocatalytic performance after 1 h of reaction based on OTP@Hexyl-TES, OTP@Octyl-TES, OTP@Decyl-TES and OTP@Dodecyl-TES systems.

To further understand the influence of the chain length of hydrophobic molecules in an interfacial microenvironment, we systematically constructed a series of triphase systems using triethylsilane (TES) with different chain lengths, including OTP@Hexyl-TES, OTP@Octyl-TES, OTP@Decyl-TES and OTP@Dodecyl-TES. Figure 4a shows the PL spectra of these four different triphase systems. It can be seen that the PL peak intensity increases with the increasing alkyl carbon chain, indicating that the long-chain hydrophobic molecules hinder the electron transfer, which will decrease the photocatalytic performance. In addition, as shown in Figure 4b, the interfacial O_2_ concentration increases as the chain length increases, but the difference is not significant when the alkyl chain exceeds eight carbons. Figure 4c shows a comparison of the dye adsorption capacity of the four films. The adsorption quantity increases with the chain length increasing due to the increased number of hydrophobic groups (such as methyl groups), which provide more sites for interaction with organic molecules. Therefore, increasing the chain length benefits the enrichment of O_2_ and organic molecules at the interface, but suppresses the interfacial electron transfer. These three factors synergistically affect the photocatalytic reaction performance. As shown in Figure 4d, when the alkyl carbon chain is relatively shorter, the low interfacial O_2_ concentration and dye adsorption limit the photooxidation performance. Conversely, when the alkyl carbon chain is long, low electron transfer efficiency decreases the performance. The OTP@Octyl-TES system exhibits the best photooxidation performance, due to the best synergistic effect of interfacial O_2_ concentration, dye adsorption capacity and electron transfer efficiency.

## 4. Conclusions

In summary, we have constructed an air–liquid–solid triphase photocatalytic system by depositing hydrophobic materials on the surface of ordered TiO_2_ porous (OTP), enabling regulation of the interfacial microenvironment through systematic variation in hydrophobic molecular chain lengths. Compared to the conventional solid–liquid diphase system (untreated OTP), the triphase architecture substantially enhances interfacial O_2_ availability and dye adsorption, thereby boosting photocatalytic performance. Our findings reveal that the chain length of the hydrophobic molecules affects the interface microenvironment significantly: increasing the chain length promotes interfacial O_2_ enrichment and dye adsorption, but suppresses electron transfer from the dye to the TiO_2_. The OTP@Octyl-TES system achieves synergistic enhancement of interfacial O_2_ concentration, dye adsorption capacity and electron transfer efficiency, leading to optimal photooxidation performance. This study establishes fundamental principles for engineering reactions in interfacial microenvironments and rationally designing high-performance catalytic systems.

## Data Availability

The original contributions presented in this study are included in the article. Further inquiries can be directed to the corresponding authors.

## Supplementary Materials

The following supporting information can be downloaded at https://www.mdpi.com/article/10.3390/biomimetics11040261/s1: Figure S1. High-resolution TEM image of the OTP@Octyl-TES film; Figure S2. EDS elemental mapping (Ti and F) images of the OTP@Octyl-TES film; Figure S3. Contact angle (CA) before and after hydrophobic layer deposition. Figure S4. (a) Comparison of photooxidation performance between OTP and OTP@Octyl-TES system. (b) The kinetics plot of the two systems; Figure S5. Absorption spectra of the RhB solutions as a function of illumination time based on OTP@Octyl-TES (a) and OTP@PDMS (b) system; Figure S6. (a) Dynamic O_2_ concentration in water after OTP@Hexyl-TES and OTP@PDMS film immersing. (b) Adsorption quantity after 1 h dark adsorption reaction based on OTP@Hexyl-TES and OTP@PDMS film.
