# Supplementary material for: Air–Liquid–Solid Triphase Interfacial Microenvironment Regulation for Efficient Visible-Light-Driven Photooxidation Based on Ordered TiO2 Porous Films
Source: Biomimetics (Basel). 2026 Apr 10;11(4):261. doi: 10.3390/biomimetics11040261 (PMC13113220; doi:10.3390/biomimetics11040261)
Supplement: Supplementary file 1 [file biomimetics-11-00261-s001.zip › biomimetics-4208162-supplementary.pdf]

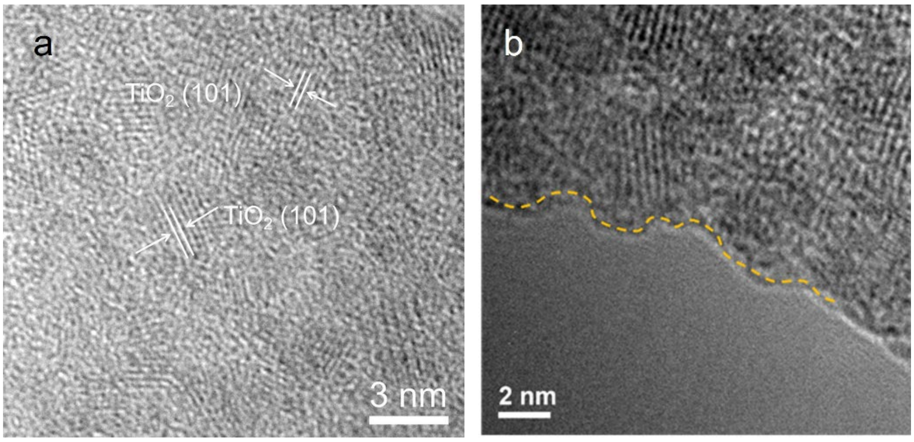

Figure S1. High resolution TEM image of the OTP@Octyl-TES film.

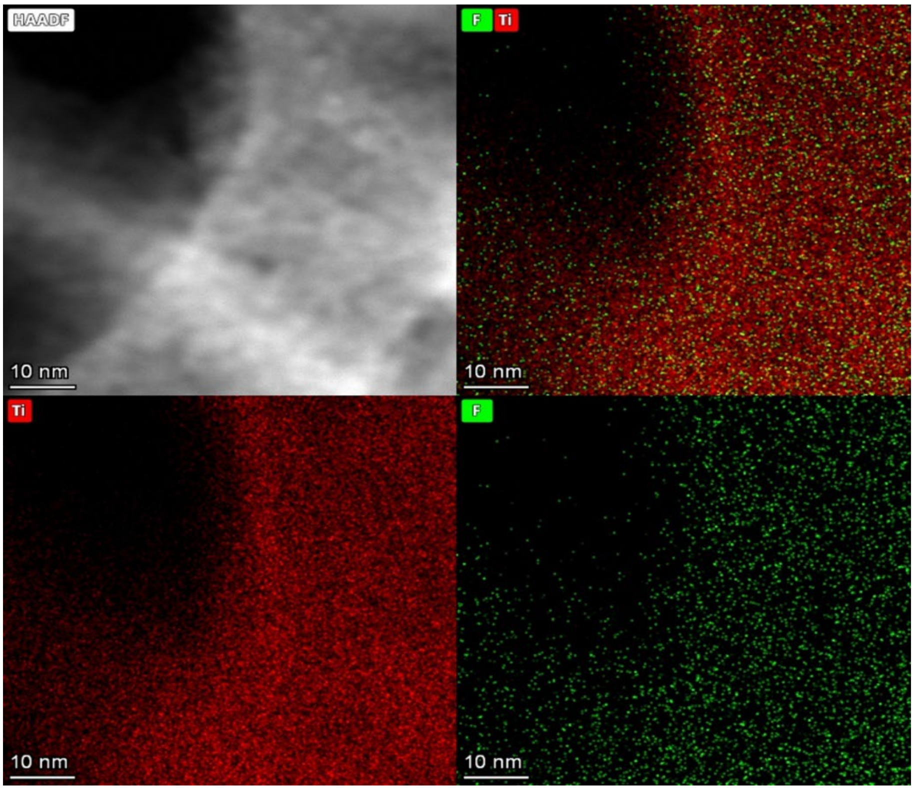

Figure S2. EDS elemental mapping (Ti and F) images of the OTP@Octyl-TES film.

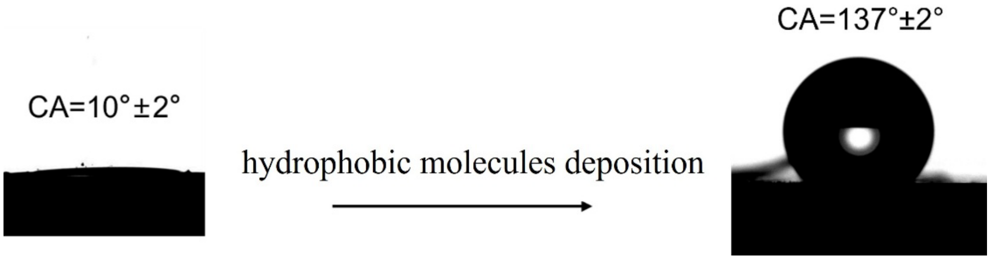

Figure S3. Contact angle (CA) before and after hydrophobic layer deposition.

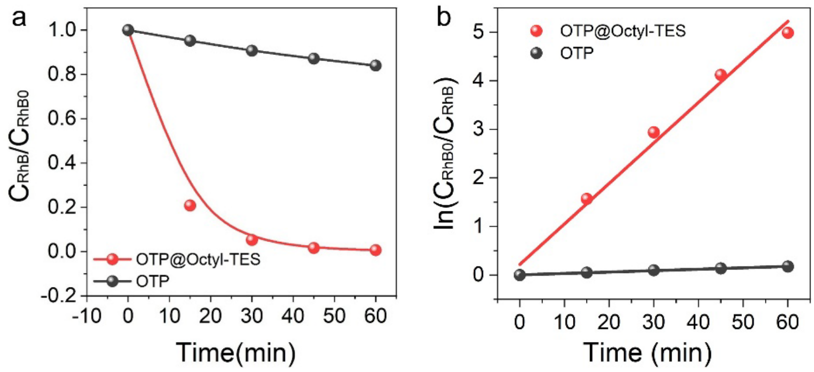

Figure S4. (a) Comparison of photooxidation performance between OTP and OTP@Octyl-TES system. (b) The kinetics plot of the two systems.

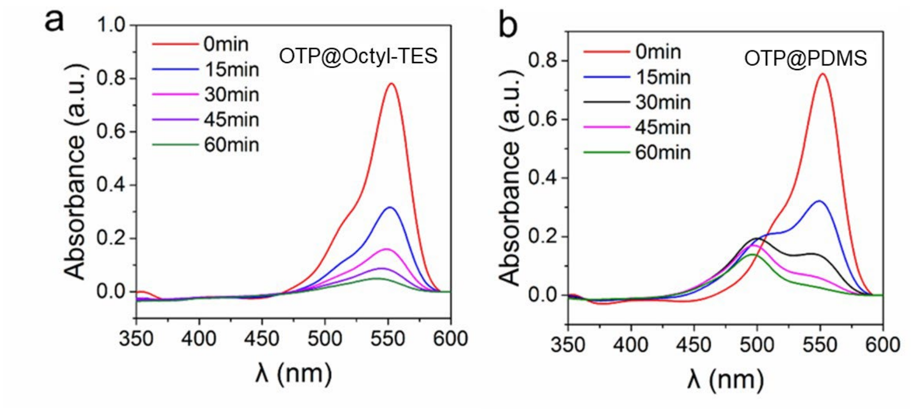

Figure S5. Absorption spectra of the RhB solutions as a function of illumination time based on OTP@Octyl-TES (a) and OTP@PDMS (b) systme.

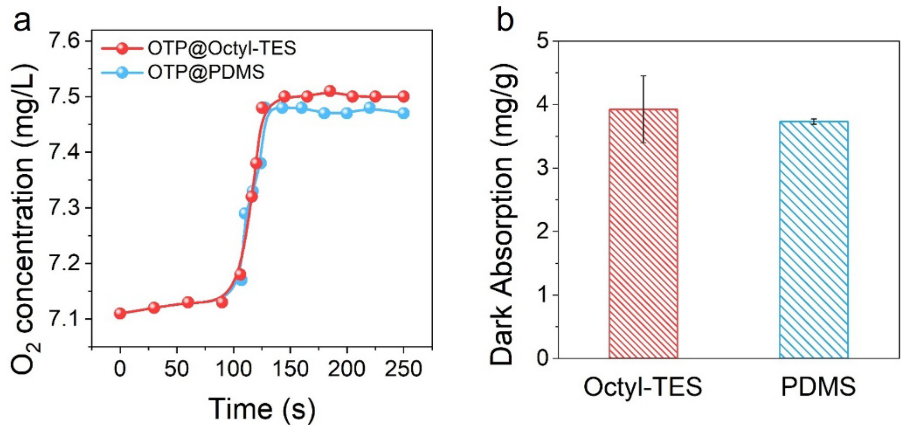

Figure S6 (a) Dynamic O_2 concentration in water after OTP@Hexyl-TES and OTP@PDMS film immersing. (b) Adsorption quantity after 1 h dark adsorption reaction based on OTP@Hexyl-TES and OTP@PDMS film.
